# Analysis of Co-Associated Transcription Factors via Ordered Adjacency Differences on Motif Distribution

**DOI:** 10.1038/srep43597

**Published:** 2017-02-27

**Authors:** Gaofeng Pan, Jijun Tang, Fei Guo

**Affiliations:** 1 School of Computer Science and Technology, Tianjin University, Tianjin, P.R. China; 2 School of Computational Science and Engineering, University of South Carolina, Columbia, USA

## Abstract

Transcription factors (TFs) binding to specific DNA sequences or motifs, are elementary to the regulation of transcription. The gene is regulated by a combination of TFs in close proximity. Analysis of co-TFs is an important problem in understanding the mechanism of transcriptional regulation. Recently, ChIP-seq in mapping TF provides a large amount of experimental data to analyze co-TFs. Several studies show that if two TFs are co-associated, the relative distance between TFs exhibits a peak-like distribution. In order to analyze co-TFs, we develop a novel method to evaluate the associated situation between TFs. We design an adjacency score based on ordered differences, which can illustrate co-TF binding affinities for motif analysis. For all candidate motifs, we calculate corresponding adjacency scores, and then list descending-order motifs. From these lists, we can find co-TFs for candidate motifs. On ChIP-seq datasets, our method obtains best AUC results on five datasets, 0.9432 for NMYC, 0.9109 for KLF4, 0.9006 for ZFX, 0.8892 for ESRRB, 0.8920 for E2F1. Our method has great stability on large sample datasets. AUC results of our method on all datasets are above 0.8.

Transcription factors (TFs) recognize specific DNA sequences near promoter regions of genes. TFs binding specificities play key roles in gene regulatory network architectures and functions^1^. TFs bind to specific DNA sequences or motifs in our genome, and interactions between TFs and DNA are elementary to the regulation of transcription. Therefore, the ability of detecting TF binding sites throughout genomes is necessary to reflect gene regulation and infer regulatory networks^2^,^3^.

Generally, the gene is not regulated by only one single TF, but instead by a combination of TFs in close proximity^4^. TFs co-localize and collaborate together, known as co-associated TFs (co-TFs) of each other. Analysis of co-TFs is an important problem in understanding the mechanism of transcriptional regulation^5^,^6^,^7^. Recently, ChIP-seq in mapping TF binding sites provide a large amount of experimental data to analyze co-TFs. There exist many technologies in mapping TF binding sites to identify some new co-TFs^8^,^9^,^10^.

Motif Enrichment Analysis (MEA) uses enrichment information of known motifs in the regions of genes to determine whether DNA-binding transcription factors have function on a set of genes^11^. There are some MEA methods for motif enrichment analysis with difference features and performances, such as ConTra^12^, PASTAA^13^, SpaMo^14^, CEAS^15^, CORE_TF^16^ and CENTDIST^17^.

ConTra (conserved TFBSs) can do motif enrichment analysis in the promoters of genes. With gene sequences of several species, ConTra checks whether motif binding sites are conserved in genes. For a list of motifs, scores reflect their binding situations to the promoter. PASTAA (Predict ASsociated Transcription factors from Annotated Affinities) uses binding affinities of TF to detect binding motifs. In PASTAA, all genes are ranked according to their predicted affinity for a given TF and their association with a given category separately. ConTra and PASTAA can only do enrichment analysis on promoter region but not genomic region.

SpaMo (spaced motif analysis) is able to infer interactions between a specific TF and TFs bound at near sites on the DNA sequence. This method can get motif spacing information facilitating the understanding of individual TF complex structures. Unlike other motif enrichment analysis method, SpaMo analyzes the enrichment of motif spacings instead of occurrences. CEAS (cis-regulatory element annotation system) can do motif finding and enrichment analysis. Given ChIPed regions and a motif, CEAS counts the number of hits, where the score of motif is greater than a cutoff, both in the ChIP region and in the whole genome, then report and rank motifs according to the binomial test P-value. CORE_TF (Conserved and Over-REpresented Transcription Factor binding sites) uses both sequence conservation-based approach and PWM approach. The combination of these two approaches can reduce false predictions when identify TF binding sites. With an input dataset, CORE_TF subsequently scans individual promoters for cross-species conservation, then employs PWM matrices. CENTDIST use the property center distribution, that two TFs are co-associated when the relative distance between them exhibits a peak-like distribution^18^,^19^,^20^. For an input ChIP-seq dataset, CENTDIST scans for the occurrence of binding sites around the peak point, then scores imbalanced distribution of motifs. SpaMo, CEAS, CORE_TF and CENTDIST can accept genomic regions as input dataset and do analysis of them.

Several studies show that if two TFs are co-associated, their ChIP-seq peaks are not only in close proximity with each other, but the relative distance of each TF with respect to another exhibits a peak-like distribution^18^,^19^,^20^. In order to analyze co-TFs, we develop a novel method to evaluate the associated situation between TFs. First, we design the sequence-specific binding score for representing patterns in biological sequences^21^,^22^. Then, we produce ordered adjacency scores based on a novel descending-order matrix. These two scores reflect the difference information between two adjacent regions to analyze the tendency of binding affinity between motif and DNA sequences. For all candidate motifs, we calculate corresponding adjacency scores, and then list descending-order motifs. From these lists, we can find co-TF binding affinities for candidate motifs. On ChIP-seq datasets, our method obtains best AUC results on five datasets, 0.9432 for NMYC, 0.9109 for KLF4, 0.9006 for ZFX, 0.8920 for ESRRB, 0.8828 for E2F1. The average performance and standard deviation of our method are better than other existing methods. Our method has great stability on large sample datasets.

## Methods

We can obtain a large amount of ChIPed TF’s location data by ChIP-seq experiment^23^. Locations of co-TFs for a particular TF always enrich around this TF’s location. One problem is to identify co-TFs of ChIPed TF with a list of ChIP-seq peaks, which map TFs on gene sequences. Assuming binding motifs of candidate co-TFs are known, the approach to this challenge is motif enrichment analysis.

In order to predict co-associated TFs, we develop a novel method to evaluate the associated situation between TFs. We design an adjacency score based on ordered adjacency differences, which can illustrate co-TF binding affinities for motif analysis.

### Sequence-Specific Binding Score

DNA motif is denoted as the conservation feature of binding sequences for one TF. We can use the common representation, Position Weight Matrix (PWM)^24^, for modeling DNA motif computationally. It has great advantages for representing patterns in biological sequences^25^.

PWM models an *l*-bases motif as a 4 × *l* matrix Θ. The entry Θ_*q,p*_ is the frequency of nucleotide 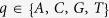 at position *p*, and all entries in each column of matrix sum to 1. Given an *l*-bases sequence *s, s*[*i*] denotes the base at position *i*, Θ_*s*[*i*],*i*_ denotes the probability of nucleotide *s*[*i*] at position *i* under PWM matrix Θ, and 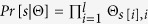 denotes the probability to produce sequence *s* from matrix Θ.

The PWM score is the log likelihood ratio of the probability *Pr*[*s*|Θ], compared to a uniform 0-markov model^26^,^27^. Given a sequence *s* and a PWM matrix Θ with length *l*, the PWM score can be defined as follows.


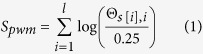


where 0.25 is the probability under uniform model.

For an *l*-bases motif, we can calculate the nucleotides sequence with maximum or minimum PWM score. The maximum PWM score can be defined as follows.


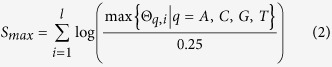


where 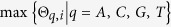 is the maximum probability chosen in each column of PWM matrix.

The minimum PWM score can be defined as follows.


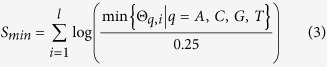


where 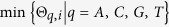 is the minimum probability chosen in each column of PWM matrix.

We can use the linear transformation to normalize each PWM score within the range of [0, 1], defined as follows.


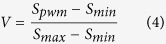


where sequence-specific binding score *V* represents the binding affinity between motif and sequences.

**Figure 1:**
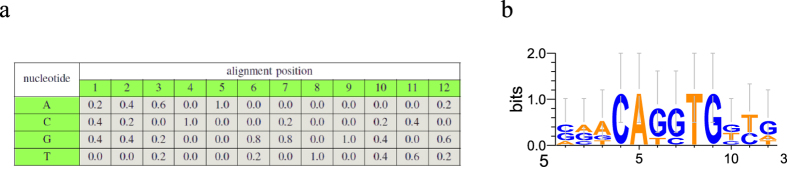
The sequence-specific binding information of motif V$MYOD_01 in TRANSFAC database. (**a**) Position Weight Matrix of V$MYOD_01. (**b**) Sequence Logo of V$MYOD_01.

The sequence-specific binding information can be shown in Fig. 1. Figure 1a is the PWM matrix of motif V$MYOD_01 in TRANSFAC database. This motif data is defined by five functional elements in three genes of mouse, and each value in this matrix represents the frequency of corresponding nucleotide at the specific position of the aligned sequences^28^. For example, 0.2 at cell (*A*, 1) means that in a set of aligned sequences, there exists 20% sequences having nucleotide *A* at position 1.

Sequence logo^29^ is a graphical representation of the sequence conservation. Figure 1b is the sequence logo of V$MYOD_01, plotted using Biopython^30^,^31^. It depicts consensus sequences and the diversity of sequences. The relative size of each letter indicates its frequency in a set of aligned sequences. We use the package of Biopython to parse TRANSFAC matrix entries and calculate the PWM score.

### Descending-Order Matrix

The peak point set in the ChIP-seq map can be represented as 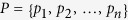. DNA sequences are extracted from the ±*m* bp region of every peak *p*_*i*_ in the ChIP-seq data, and the sequence set is constructed as 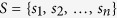.

For each motif, we scan all sub-sequences in the DNA sequence set *S* with the PWM matrix Θ, and obtain 2 × *m* scores for each DNA sequence. We partition each DNA sequence into *b* bins with respect to the distance from the peak point, where each bin is of size 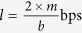. In each bin, we sort *r* = *l* × *n* normalized PWM scores in descending-order, then construct a *r* × *b* matrix *M*_*s*_ in which every column contains sorted scores of corresponding bin.

**Figure 2:**
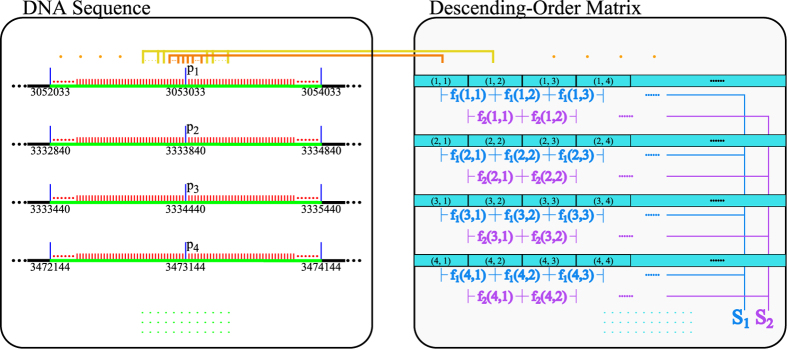
Extracting sequences and creating matrix *M*_*s*_ on NANOG dataset.

We analyze an example of extracting sequences and creating matrix *M*_*s*_ on NANOG (GSM288345) dataset^32^, shown in Fig. 2. Figure 2 (left) represents the sequence set extracted from mouse genome using ChIP-seq of NANOG. The middle point is corresponding to the peak point, and the sequence is 2000 bps length including both left and right 1000 bps regions. The position 3053033 is a peak point on NANOG, and the sequence [3052033, 3054033] is extracted as the first sequence. Figure 2 (right) represents the matrix of normalized binding scores, with the descending-order column.

### First-Order Adjacency Difference

We calculate the difference between two adjacent columns in matrix *M*_*s*_, to analyzing the tendency of binding affinity between motif and sub-sequences. We extract a pair of adjacent columns in each region, and calculate first-order adjacency difference *f*_1_ between each pair of adjacent cells in the same row, defined as follows.


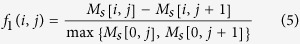


where *i* is the index of each row, and *j* and *j* + 1 are indices of columns. *M*_*s*_[0, *j*] and *M*_*s*_[0, *j* + 1] are maximum values of *j*-th and (*j* + 1)-th columns, since *M*_*s*_ is descending-order matrix.

Considering co-TFs distribution^18^,^19^,^20^, they always appear near around peak points. Therefore, we use a gamma distribution function^33^ to weight the *f*_1_ score, which has large values at near regions and small values at remote regions.

We sum *f*_1_ values in each region and weight results by the gamma distribution *g(j*|*c, γ*) according to the region *j*. Then, the total score of first-order adjacency difference can be defined as follows.


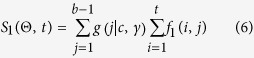


### Second-Order Adjacency Difference

When defining above scores, we only consider equal possibility model as the background model. However, CG/AT bias around ChIP-seq peak points has unbalanced distribution. We define second-order adjacency difference *f*_2_ to reduce the effect of unbalanced CG/AT bias noise, as follows.


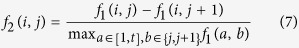


where *f*_1_ values can be calculated using equation 5. The denominator in the fraction is maximum value of all *f*_1_ values of *j*-th and (*j* + 1)-th columns.

A large or positive difference means a dense-binding region, but a small or negative difference means a sparse-binding region. Therefore, we sum *f*_2_ values in each region and use sigmoid function^34^ to normalize the difference of each region. Then, the total score of second-order adjacency difference can be defined as follows.


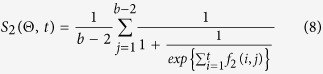


### Adjacency Score for Motif Analysis

The final scoring function is the combination of above two difference scores. For a motif Θ, we can calculate the final adjacency score for motif analysis around ChIPed points, defined as follows.


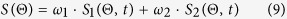


where the parameter *t* can be calculated to maximize *S*_1_(Θ, *t*) for each motif Θ; *ω*_1_ and *ω*_2_ are chosen according to the contribution of first-order adjacency difference and second-order adjacency difference to the final adjacency score. We list possible *ω*_1_ and *ω*_2_ values and their effects on dataset NMYC in Supplementary Table S3.

For all candidate motifs, we calculate corresponding adjacency scores for each dataset, and then list descending-order motifs corresponding to their motif scores. From these scores, we can find co-TF binding affinities for candidate motifs.

### Data Availability

Codes, datasets and results are available for download from https://figshare.com/s/3966b4cdcac5caaaa0d8.

## Results

We apply our method on several datasets, and use AUC to evaluate the performance. Then, we analyze the ordered adjacency difference defined by our method. Finally, we compare results of our method with other existing methods, and find that our method improves on some datasets.

### Data Set

We use ChIP-seq map of TFs, genome sequence and motif matrix to analyze co-TFs. ChIP-seq data^32^ are mapping of 13 transcription factors in mouse embryonic stem (ES) cells, shown in Supplementary Table S1. We test on 13 transcription factors, such as Nanog, Oct4, STAT3, Smad1, Sox2, Zfx, c-Myc, n-Myc, Klf4, Esrrb, Tcfcp2l1, E2f1 and p300. Among these factors, p300 is transcription regulator and others are sequence specific transcription factors. From these TFs data, we use the chromosome number and peak location in mouse (Mus musculus) genome.

TRANSFAC^35^ provides data on eukaryotic transcription factors, their experimentally-proven binding sites, consensus binding sequences (PWMs) and regulated genes. The nucleotide distribution matrix of aligned binding sequences is provided in the TRANSFAC matrix. In the public version database, 398 matrices can be grouped into six categories as vertebrates, insects, plants, fungi, nematodes and bacteria, and 292 of them are vertebrates used by our method. The mouse genome GRCm38^36^ is used to extract sub-sequences corresponding to peak locations from ChIP-seq data.

### Area Under Curve

For analyzing our method, we use the area under receiver operating characteristic (ROC) curve (AUC)^37^ to evaluate our results. In the ROC graph, the curve is created by plotting TPR against FPR at various threshold settings^38^. Higher AUC value means that the classifier is scoring a positive instance greater than a negative instance, in other words that this classifier is more efficient and accurate.

Our method produces a motif score for each candidate vertebrate motif in TRANSFAC database, and uses the ROC curve to evaluate the performance of scoring motifs. We group a ranked list of vertebrate TRANSFAC motifs corresponding to their factor families. All vertebrate motifs in TRANSFAC database can be divided into the positive set and the negative set, based on the current ChIP-seq data. Then, we can plot the ROC curve using ranked list of motif families and calculate the AUC value. Using AUC results, we can evaluate our method and compare to other methods.

### Assessment of Ordered Adjacency Difference

**Figure 3:**
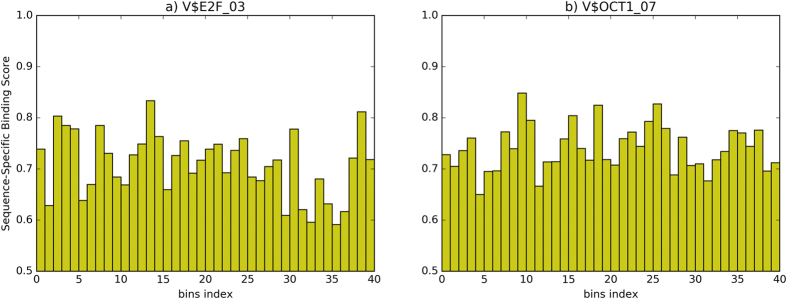
Comparison of sequence-specific binding scores for two motifs on c-Myc. (**a**) sequence-specific binding scores in each bin of motif V$E2F_03. (**b**) sequence-specific binding scores in each bin of motif V$OCT1_07.

DNA motifs have different sequence-specific binding scores around ChIPed peak points. On a specific ChIP-seq dataset, if a candidate motif is a co-TF, it would enrich at the near area and disperse at the remote area. We compare different distributions of sequence-specific binding scores for two motifs on c-Myc, as shown in Fig. 3. The motif V$E2F_03 (Fig. 3a) has good enrichment on 0 to 30 bins, and sequence-specific binding scores of the near area are much higher than the remote area. The motif V$OCT1_07 (Fig. 3b) does not have significant changes between the near area and the remote area.

**Figure 4:**
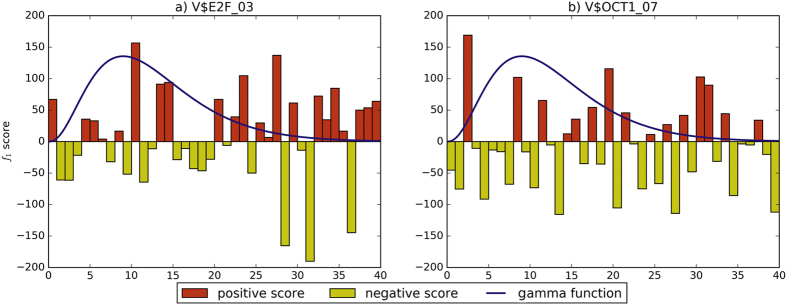
Comparison of *f*_1_ scores for two motifs on c-Myc.

We use gamma distribution to weight the *f*_1_ score, which can enlarge scores at specific ranges near the origin and shrink scores at the remote ranges. We compare different distributions of *f*_1_ scores for two motifs on c-Myc, as shown in Fig. 4. V$E2F_03 (Fig. 4a) is a co-TF on c-Myc, having clear boundary between regions 0–15 and regions 15–40. Positive scores locate in regions 0–15 that gamma distribution values are large, and negative scores are not too large to effect changes. V$OCT1_07 (Fig. 4b) are almost similar in all regions, and large negative scores reduce the effect of changes between enrichment regions and remote regions.

**Figure 5:**
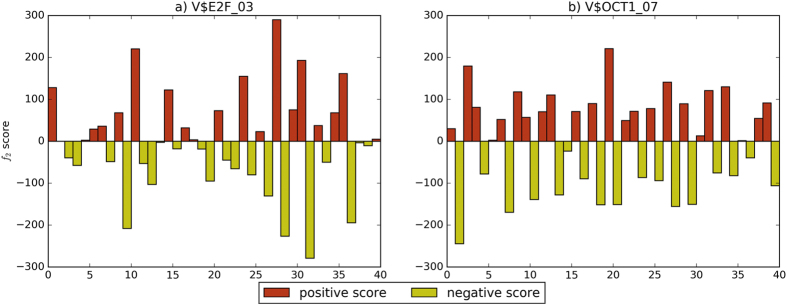
Comparison of *f*_2_ scores for two motifs on c-Myc.

In order to reflect distribution of the *f*_1_ score, positive changes enrich motif binding affinity, and negative changes lead to opposite situation. We also compare different distributions of all *f*_2_ scores for two motifs on c-Myc, as shown in Fig. 5. V$E2F_03 (Fig. 5a) has more positive scores than negative scores, which enhance binding ability. V$OCT1_07 (Fig. 5b) has large negative scores in all regions.

### Comparison to Existing Methods

We evaluate the performance of our method on ChIP-seq data in ES cells. Also, we compare to other three existing methods having good performance on classifying co-TFs, CEAS^15^, CORE_TF^16^ and CENTDIST^17^. The performance result can be accessed from the AUC result in Supplementary Table S2 and Supplementary Fig. S1.

CEAS is a web server that can identify enriched transcription factor-binding motifs from user-defined genome-scale ChIP regions. CEAS uses several features, including sequence retrieval, conservation plot, nearby gene mapping, motif finding and enrichment analysis. CORE_TF can identify common transcription factor binding sites in promoters of co-regulated genes. CORE_TF finds experimental datasets for over represented PWMs from TRANSFAC database, and a unique feature matchs the random set to the experimental set of promoters by GC content. CENTDIST is a web based co-motif scanning program. It does not need user specific background and parameters being automatically determined on the motif distribution around ChIP-seq peaks.

**Figure 6:**
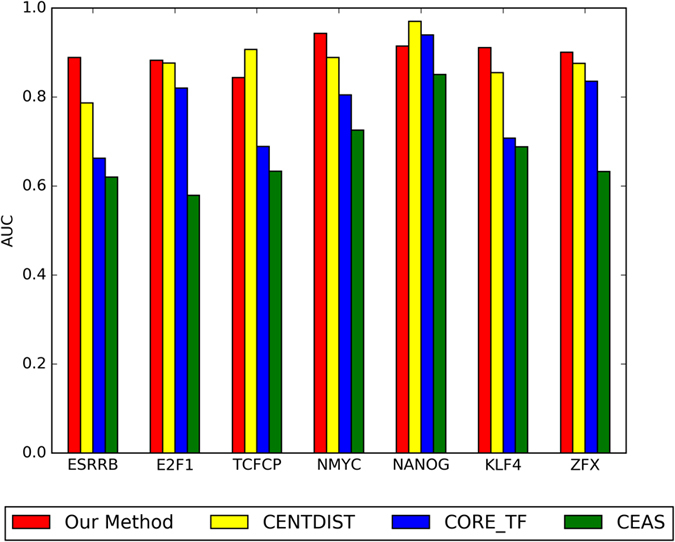
Comparison of our method, CENTDIST, CEAS, and CORE_TF on seven large sample ChIP-seq datasets in ES cells.

Our method has great stability on large sample datasets. When the size of dataset increases, our method can archive better result than other methods, such as CENTDIST, CORE_TF and CEAS. Existing methods can’t keep their effectiveness on large sample datasets. We compared the performance of our method on seven large sample ChIP-seq datasets to these existing methods. Comparing to existing methods, the performance of our method on seven datasets is shown in Fig. 6.

In Table 1, our method is applied on three datasets with size more than 20000 peak points, such as ESSRRB, E2F1 and TCFCP. On ESRRB dataset, our method achieves 0.8892 AUC value, improving accuracy at least by 0.1023. On E2F1 dataset, our method achieves 0.8920 AUC value, improving accuracy at least by 0.0159. On TCFCP dataset, our method achieves 0.8437 AUC value, less than result of CENTDIST (0.9072).

In Table 2, our method is applied on four datasets with size more than 5000 peak points and less than 20000 peak points, such as NMYC, NANOG, KLF4 and ZFX. On NMYC dataset, our method achieves 0.9432 AUC value, improving accuracy at least by 0.0543. On KLF4 dataset, our method achieves 0.9109 AUC value, improving accuracy at least by 0.0559. On ZFX dataset, our method achieves 0.9006 AUC value, improving accuracy at least by 0.0248. On NANOG dataset, our method achieves 0.9148 AUC value, less than result of CENTDIST (0.9699).

In order to evaluate the stability of our method, we compute average value and standard deviation of AUC results for our method, as shown in Table 3. On three datasets with size more than 20000 peak points, the mean value of AUC results by our method is 0.8750 and the standard deviation is 0.0271. On four datasets with size more than 5000 peak points and less than 20000 peak points, the mean value of AUC results by our method is 0.9174 and the standard deviation is 0.0182. On all seven datasets with size more than 5000 peak points, the mean value of AUC results by our method is 0.8992 and the standard deviation is 0.0304. The mean value of our method is greater than other existing methods, and the standard deviations of our method is the smallest one.

**Figure 7:**
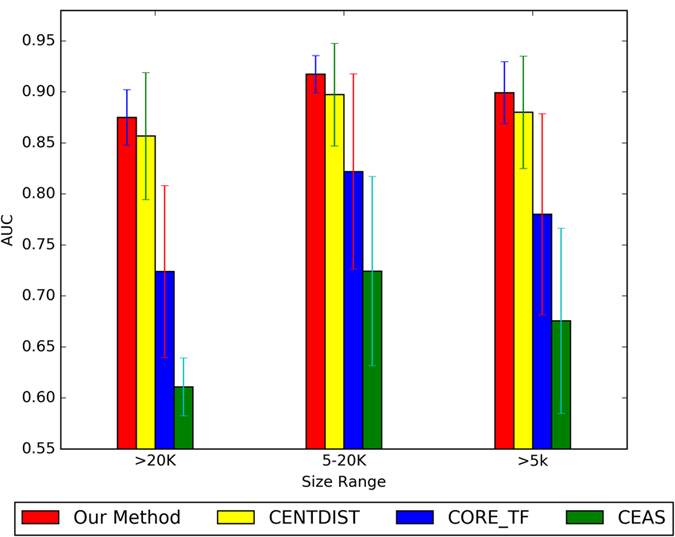
Evaluate the stability of our method, CENTDIST, CEAS, and CORE_TF on seven ChIP-seq datasets.

In Fig. 7, we can see that both the average performance and standard deviation of our method are better than other existing methods.

## Conclusion

In order to analyze co-associated TFs, we develop a novel method to evaluate the associated situation between TFs. We design an adjacency score based on ordered adjacency differences, which can illustrate co-TF binding affinity for motif analysis. Our method obtains best AUC results on five datasets, 0.9432 for NMYC, 0.9109 for KLF4, 0.9006 for ZFX, 0.8892 for ESRRB, 0.8920 for E2F1. AUC results of our method on all datasets are above 0.8.

## Additional Information

**How to cite this article:** Pan, G. *et al*. Analysis of Co-Associated Transcription Factors via Ordered Adjacency Differences on Motif Distribution. *Sci. Rep.* **7**, 43597; doi: 10.1038/srep43597 (2017).

**Publisher's note:** Springer Nature remains neutral with regard to jurisdictional claims in published maps and institutional affiliations.

## Supplementary Material

Supplementary Tables and Figures

## Figures and Tables

**Table 1:** AUC results of our method, CENTDIST, CEAS, and CORE_TF on three ChIP-seq datasets with size more than 20000 peak points.

	Our method	CENTDIST	CORE_TF promBG^a^			CORE_TF randBG^b^			CEAS^c^		
			200	400	1000	200	400	1000	200	400	1000
ESRRB	**0**.**8892**	0.7869	0.6373	0.6627	0.6065	0.5359	0.5451	0.6183	0.6203	0.6072	0.6111
E2F1	**0**.**8828**	0.8761	0.8202	0.7966	0.7758	0.8076	0.7862	0.7303	0.5789	0.5625	0.5746
TCFCP	0.8437	**0**.**9072**	0.6889	0.6719	0.5386	0.6627	0.6484	0.6641	0.6333	0.6144	0.6105

^a^CORE_TF promBG under promoter background uses enriched regions with size 200, 400 and 1000.

^b^CORE_TF randBG under random genome background uses enriched regions with size 200, 400 and 1000.

^c^CEAS uses enriched regions with size 200, 400 and 1000.

**Table 2:** AUC results of our method, CENTDIST, CEAS, and CORE_TF on four ChIP-seq datasets with size more than 5000 peak points and less than 20000 peak points.

	Our method	CENTDIST	CORE_TF promBG^a^			CORE_TF randBG^b^			CEAS^c^		
			200	400	1000	200	400	1000	200	400	1000
NMYC	**0**.**9432**	0.8889	0.8052	0.7915	0.7627	0.7922	0.7719	0.7418	0.7255	0.6137	0.6039
NANOG	0.9148	**0**.**9699**	0.9320	0.9399	0.9020	0.9255	0.9046	0.8327	0.8386	0.8510	0.7268
KLF4	**0**.**9109**	0.8550	0.7075	0.7058	0.6908	0.7058	0.6950	0.6813	0.6708	0.6883	0.6021
ZFX	**0**.**9006**	0.8758	0.8353	0.8248	0.7732	0.8288	0.8013	0.7190	0.6327	0.5137	0.5137

^a^CORE_TF promBG under promoter background uses enriched regions with size 200, 400 and 1000.

^b^CORE_TF randBG under random genome background uses enriched regions with size 200, 400 and 1000.

^c^CEAS uses enriched regions with size 200, 400 and 1000.

**Table 3:** Evaluate on AUC values of our method, CENTDIST, CEAS, and CORE_TF on seven ChIP-seq datasets.

				CORE_TF promBG			CORE_TF randBG			CEAS		
		Our method	CENTDIST	200	400	1000	200	400	1000	200	400	1000
[20000, ∞)	*μ*	**0**.**8719**	0.8567	0.7155	0.7104	0.6403	0.6687	0.6599	0.6709	0.6108	0.5947	0.5987
	*σ*	**0**.**0246**	0.0624	0.0943	0.0748	0.1222	0.1360	0.1210	0.0563	0.0284	0.0281	0.0209
[5000, 20000]	*μ*	**0**.**9174**	0.8974	0.8200	0.8155	0.7822	0.8131	0.7932	0.7437	0.7169	0.6667	0.6116
	*σ*	**0**.**0182**	0.0503	0.0925	0.0969	0.0879	0.0910	0.0867	0.0644	0.0896	0.1422	0.0876
[5000, ∞)	*μ*	**0**.**8992**	0.8800	0.7752	0.7705	0.7214	0.7512	0.7361	0.7125	0.6714	0.6358	0.6061
	*σ*	**0**.**0304**	0.0551	0.1018	0.0986	0.1208	0.1275	0.1171	0.0681	0.0866	0.1089	0.0635
