# Supplementary material for: Analysis of Co-Associated Transcription Factors via Ordered Adjacency Differences on Motif Distribution
Source: Sci Rep. 2017 Feb 27;7:43597. doi: 10.1038/srep43597 (PMC5327392; doi:10.1038/srep43597)
Supplement: Supplementary Tables and Figures [file srep43597-s1.pdf]

Analysis of Co-Associated Transcription Factors via Ordered Adjacency Differences on Motif Distribution Supplementary Tables and Figures

Gaofeng Pan[1], Jijun Tang[1, 2], and Fei Guo[1, *]

[1]School of Computer Science and Technology, Tianjin University, Tianjin, P.R.China.
[2]School of Computational Science and Engineering, University of South Carolina, Columbia, USA
[*]corresponding author fguo@tju.edu.cn

January 3, 2017

Table S1: Motif family of TFs and co-TFs for ChIP-seq data.

**ChIP-Seq Data**	**Motif Family of TFs and co-TFs**	**Size**	**GSM Code**
Nanog	ERE, NANOG, OCT, SOX, STAT	10343	GSM288345
Oct4	CP2, E2F, EBOX, ERE, NANOG, OCT, SOX, STAT	3761	GSM288346
Sox2	ERE, CP2, NANOG, OCT, SOX, STAT	4526	GSM288347
Smad1	ERE, CP2, NANOG, OCT, SOX, STAT	1126	GSM288348
E2f1	CP2, E2F, EBOX, OCT, STAT, ZF5	20699	GSM288349
Tcfcp2l1	CP2, E2F, OCT, SOX, STAT	26910	GSM288350
Zfx	CP2, E2F, EBOX, OCT, ZF5	10338	GSM288352
Stat3	E2F, EBOX, ERE, CP2, NANOG, OCT, SOX, STAT	2546	GSM288353
Klf4	E2F, EBOX, ERE, NANOG, OCT, SOX, STAT, ZF5	10875	GSM288354
Esrrb	ERE, NANOG, OCT, SOX, STAT	21647	GSM288355
c-Myc	E2F, EBOX, ZF5	3422	GSM288356
n-Myc	E2F, EBOX, OCT, STAT, ZF5	7182	GSM288357
p300	ERE, CP2, NANOG, OCT, SOX, STAT	524	GSM288359

Table S2: AUC results of our method, CENTDIST, CEAS, and CORE_TF on 13 ChIP-seq datasets

	our method	CENTDIST	CORE_TF promBG [a]			CORE_TF randBG [b]			CEAS [c]		
			200	400	1000	200	400	1000	200	400	1000
CMYC	0.9556	**0.9957**	0.9892	0.9742	0.9355	0.9742	0.9505	0.9097	0.7731	0.7828	0.5806
E2F1	**0.8920**	0.8761	0.8202	0.7966	0.7758	0.8076	0.7862	0.7303	0.5789	0.5625	0.5746
ESRRB	**0.8892**	0.7869	0.6373	0.6627	0.6065	0.5359	0.5451	0.6183	0.6203	0.6072	0.6111
KLF4	**0.9109**	0.8550	0.7075	0.7058	0.6908	0.7058	0.6950	0.6813	0.6708	0.6883	0.6021
NANOG	0.9148	**0.9699**	0.9320	0.9399	0.9020	0.9255	0.9046	0.8327	0.8386	0.8510	0.7268
NMYC	**0.9432**	0.8889	0.8052	0.7915	0.7627	0.7922	0.7719	0.7418	0.7255	0.6137	0.6039
OCT4	0.8000	**0.9300**	0.8767	0.8908	0.9067	0.8625	0.8342	0.7900	0.8650	0.8175	0.8017
P300	0.8621	0.8646	**0.9397**	0.9364	0.8657	0.8860	0.8169	0.7270	0.7917	0.7741	0.6184
SMAD1	0.8805	**0.9507**	0.9430	0.9287	0.8520	0.9364	0.9167	0.8191	0.7906	0.8531	0.7007
SOX2	0.8644	**0.9507**	0.9035	0.9068	0.8947	0.9145	0.8969	0.8235	0.8531	0.8448	0.8684
STAT3	0.8017	**0.9175**	0.8742	0.8525	0.7875	0.7892	0.7275	0.7300	0.8067	0.7513	0.7546
TCFCP	0.8437	**0.9072**	0.6889	0.6719	0.5386	0.6627	0.6484	0.6641	0.6333	0.6144	0.6105
ZFX	**0.9006**	0.8758	0.8353	0.8248	0.7732	0.8288	0.8013	0.7190	0.6327	0.5137	0.5137

[a] CORE_TF promBG under promoter background uses enriched regions with size 200, 400 and 1000;
[b] CORE_TF randBG under random genome background uses enriched regions with size 200, 400 and 1000;
[c] CEAS uses enriched regions with size 200, 400 and 1000.

Table S3: Different parameters and the corresponding effects on dataset NMYC

ω_1	ω_2	AUC of Adjacency Scores
0.1	0.9	0.7358
0.2	0.8	0.7983
0.3	0.7	0.8636
0.4	0.6	0.8920
0.5	0.5	0.9034
0.6	0.4	0.9176
0.7	0.3	0.9290
0.8	0.2	0.9375
**0.9**	**0.1**	**0.9432**

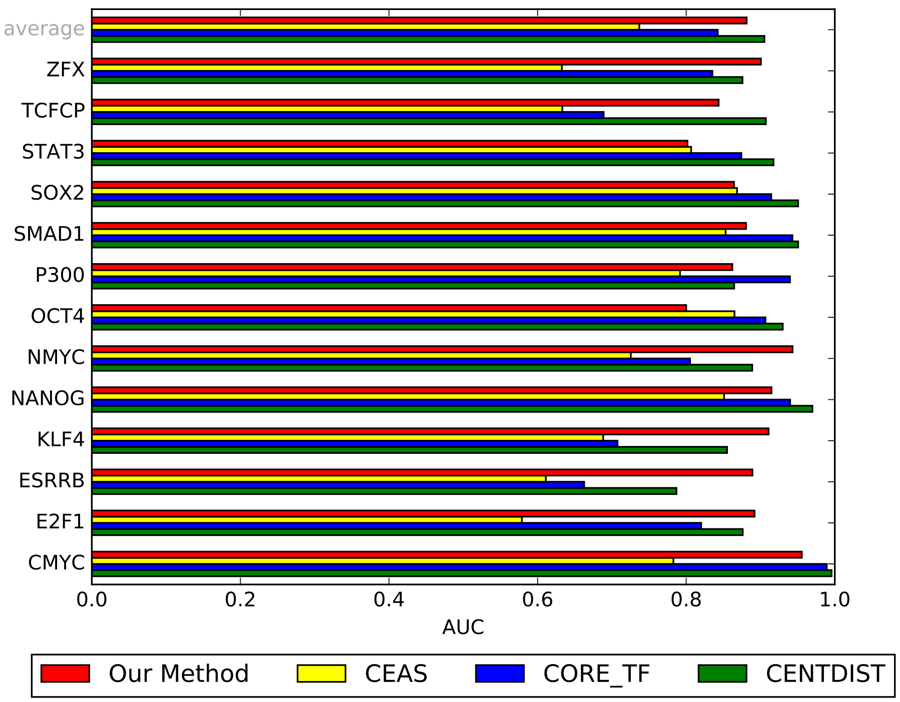

Figure S1: Comparison of our method, CENTDIST, CEAS, and CORE_TF on ChIP-seq data in ES cells.
